# Successful treatment with radiation therapy for desmoid-type fibromatosis with unilateral hydronephrosis: a case report

**DOI:** 10.1186/s13256-021-03088-7

**Published:** 2021-10-27

**Authors:** Yojiro Ishikawa, Rei Umezawa, Takaya Yamamoto, Noriyoshi Takahashi, Kazuya Takeda, Yu Suzuki, Keiichi Jingu

**Affiliations:** Department of Radiation Oncology, Tohoku University Graduate School of Medicine, 1-1 Seiryo-chou, Aoba-ku, Sendai, 980-8574 Japan

**Keywords:** Aggressive fibromatosis, Desmoid-type fibromatosis, Hydronephrosis, Radiation therapy

## Abstract

**Background:**

Desmoid-type fibromatosis is a rare disease that can result in hydronephrosis. Hydronephrosis associated with desmoid-type fibromatosis often requires surgery or ureteral stent insertion. Although radiation therapy is recommended for inoperable cases of desmoid-type fibromatosis, there has been no report of treatment for hydronephrosis associated with desmoid-type fibromatosis by radiation therapy alone. We herein report a case of successful treatment for inoperable recurrence of desmoid-type fibromatosis with unilateral hydronephrosis by radiation therapy alone.

**Case presentation:**

A 43-year-old Japanese female underwent resection of desmoid-type fibromatosis in the right inguinal region and combined resection of the right external iliac vein 5 years before. Other treatment was not performed because of her pregnancy. Four years after surgery, desmoid-type fibromatosis recurred in the right pelvic wall. Cyclooxygenase-2 selective inhibitor treatment was given for 1 year, but her desmoid-type fibromatosis enlarged to more than 10 cm, and she had swelling of her right leg and hydronephrosis of her right kidney. The patient received 50.4 Gy in 28 fractions of prophylactic irradiation using 10 MV X-ray and 9 Gy in five fractions of a sequential boost for the recurrent desmoid-type fibromatosis. Although there was temporary tumor progression at 1 month after radiation therapy, slow regression of the tumor was seen. At 5 years after radiation therapy, there was no disease progression or severe complications.

**Conclusion:**

We experienced successful treatment for an inoperable case of desmoid-type fibromatosis with hydronephrosis. Moderate-dose radiation therapy alone is an effective and feasible approach for the management of hydronephrosis associated with desmoid-type fibromatosis.

## Background

Desmoid-type fibromatosis (DTF) is a rare disease that occurs in 2–4 people per million people annually [1, 2]. Abdominal wall DTF is especially common in females of childbearing age, and the highest incidence is in people between the ages of 10 and 40 years [3]. DTF can be asymptomatic or can result in pain, swelling, hydronephrosis, neurovascular damage, and other complications [4].

Wide resection is required for treating DTF in some cases, but recurrence is frequent [5]. Radiation therapy (RT) is a useful treatment for postoperative or inoperable DTF. In addition, there are some reports of irradiation for DTF being performed by proton beam or carbon ion radiation therapy [6, 7]. Although more conservative management has recently been recommended [8], treatment for a patient who has recurrence of DTF after surgery or a patient for whom conservative treatment cannot be continued due to complications such as hydronephrosis is not clear.

## Case presentation

**Fig. 1:**
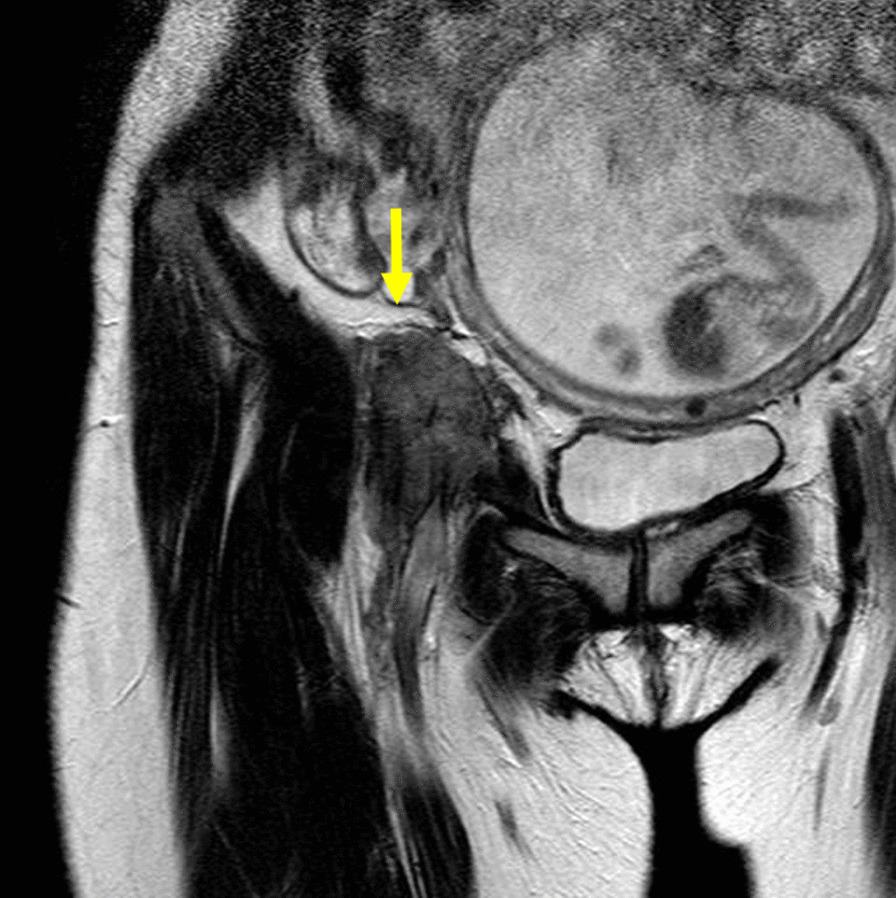
Coronal T2-weighted magnetic resonance imaging of the pelvis. The lesion in the right inguinal region shows iso- to slightly high intensity (yellow arrow)

**Fig. 2:**
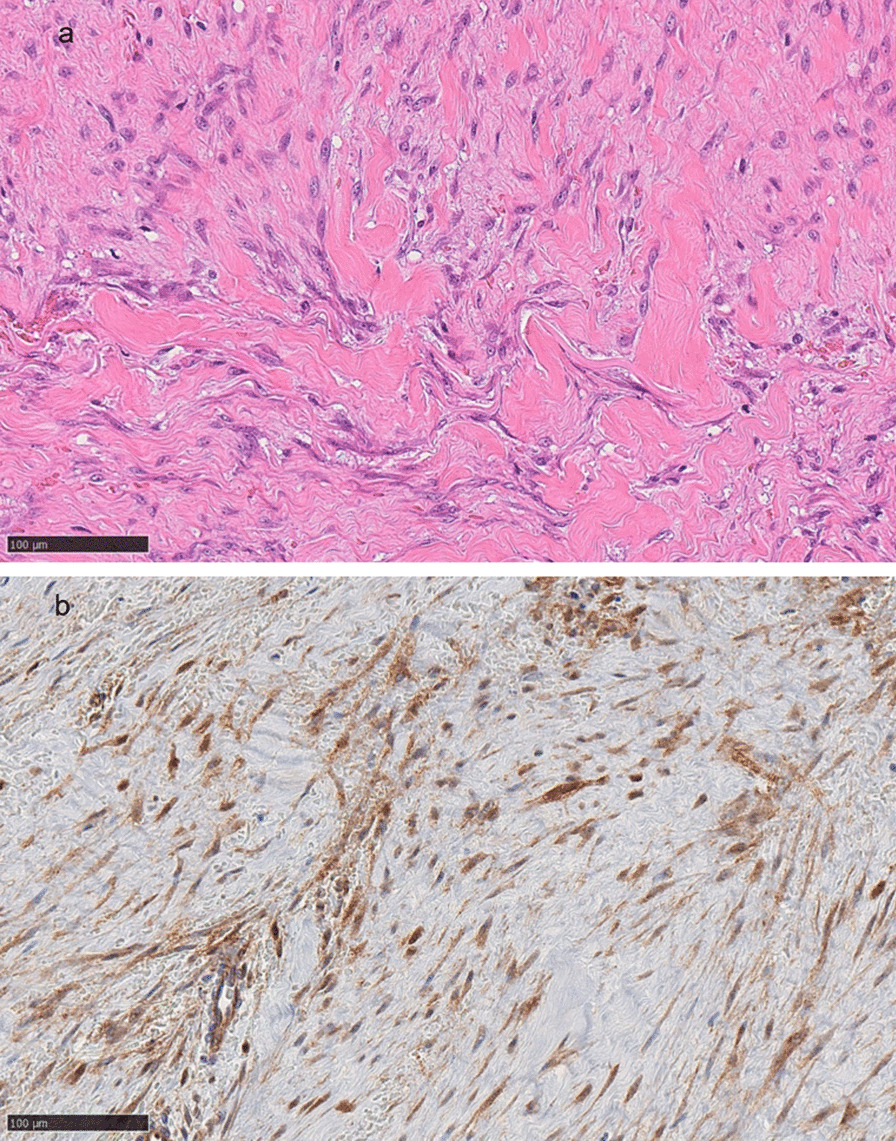
**a** Histopathology showing proliferation of spindle-shaped cells surrounded by collagen (hematoxylin and eosin stain; original magnification ×200). **b** Immunohistochemical staining showing positivity for intranuclear β-catenin (β-catenin; original magnification ×200)

A 38-year-old Japanese female in the 15th week of pregnancy presented with a dull pain in her right inguinal region. She had no medical history and no family history of familial adenomatous polyposis. Magnetic resonance imaging (MRI) revealed a tumor around the external iliac artery to the femoral artery just below the right abdominal wall (Fig. 1). Fine needle aspiration biopsy was performed, and the findings were suggestive of DTF. In the 18th week of pregnancy, resection of the DTF was performed. Surgical biopsies of the tumor revealed a DTF and suggested a positive margin of the pelvic wall. Immunostaining was positive for β-catenin and negative for estrogen receptors and progesterone receptors (Fig. 2). RT after surgery was not possible because the patient was pregnant.

**Fig. 3:**
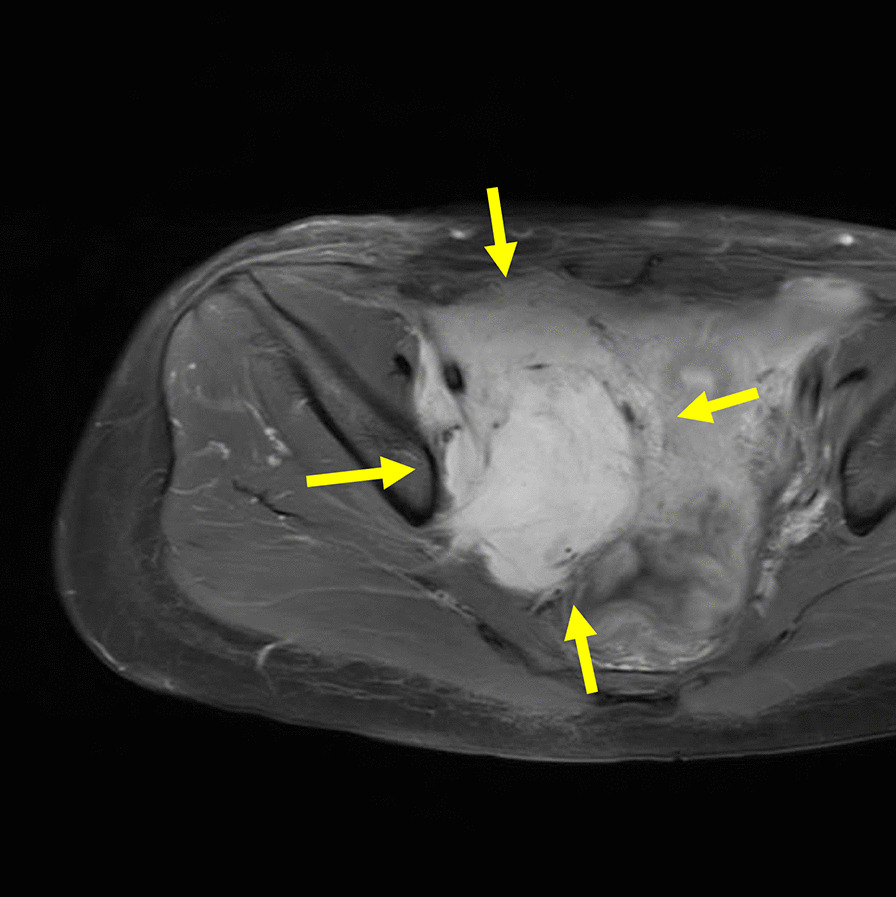
Post-gadolinium axial magnetic resonance imaging of the pelvis showing strong and homogeneous enhancement of lesion by intravenous administration of gadolinium (yellow arrow)

**Fig. 4:**
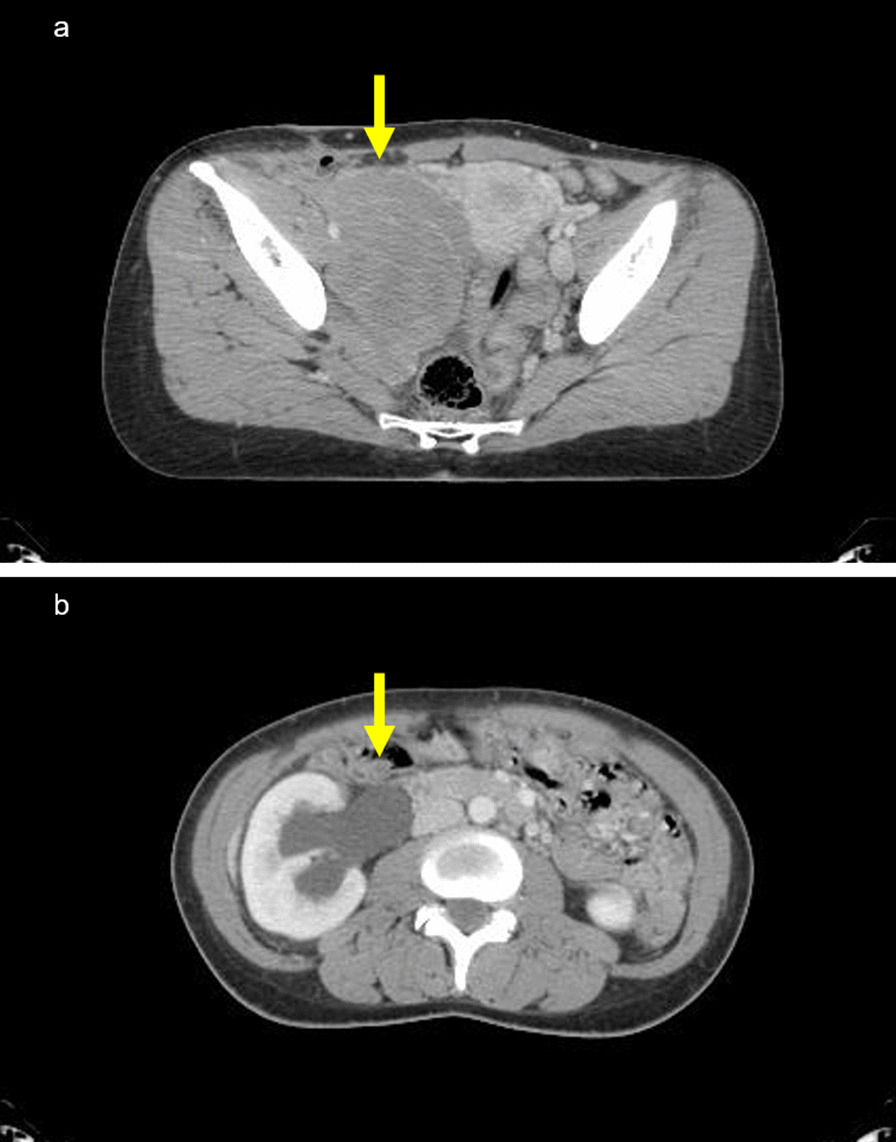
**a** Axial enhanced computed tomography scan image of the pelvis. The lesion is located close to the iliac artery, uterus, and rectum (yellow arrow). **b** Axial enhanced computed tomography scan image of the abdomen revealing hydronephrosis of the right kidney. The right kidney and renal pelvis are enlarged. The right renal parenchyma is mildly thin (yellow arrow)

At 3 years after surgery, marginal recurrence of DTF in the right pelvic wall was diagnosed by MRI. Cyclooxygenase-2 selective inhibitor treatment was given for 1 year. However, MRI showed enlargement of the recurrent tumor measuring 6.8 × 10.0 cm in size (Fig. 3), and swelling of her right leg occurred. Computed tomography showed severe right hydronephrosis (Fig. 4). Her laboratory values included a serum creatinine level of 0.75 mg/dL. Surgeons recommended ureteral stent insertion for right hydronephrosis. However, she rejected stent insertion owing to concerns about the risks associated with stenting. Surgeons also recommended RT for recurrent DTF because it was located close to the right internal iliac artery and nerve.

**Fig. 5:**
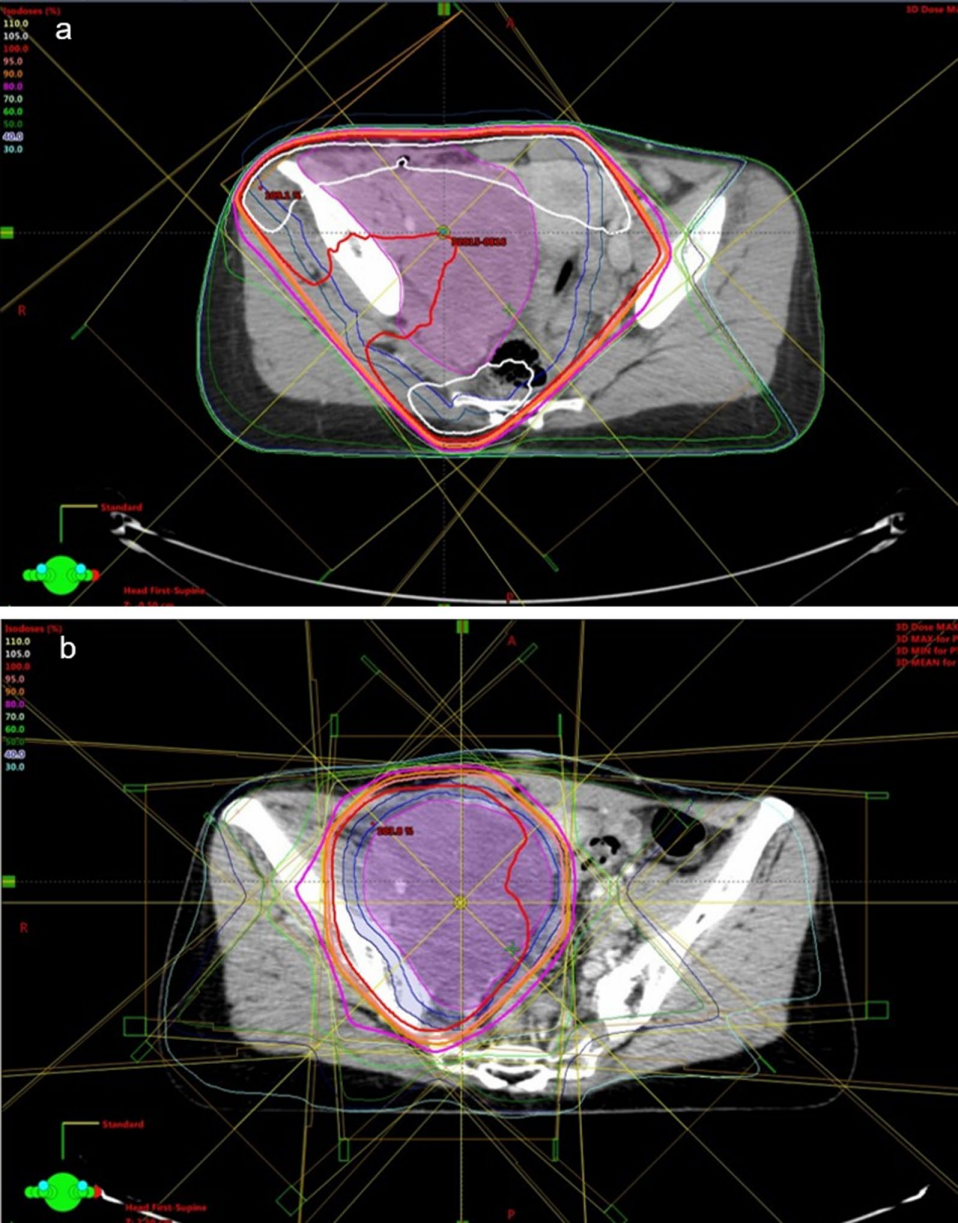
**a** Dose distribution of radiotherapy with 50.4 Gy by four fields. **b** Dose distribution of boost radiotherapy with 9 Gy by eight fields

Definitive RT was performed using 10 MV X-ray. The patient received 50.4 Gy in 28 fractions of prophylactic irradiation for the right inguinal region of the postoperative lesion with a margin of 5 cm added to macroscopic tumors. After initial irradiation of 50.4 Gy, the patient received 9 Gy in five fractions of a sequential boost for the right pelvic wall tumor to a total dose of 59.4 Gy (Fig. 5). Acute perianal dermatitis of grade 2 occurred at the end of RT, and it could be controlled with conservative therapy. RT did not induce any deterioration in renal function or degree of hydronephrosis.

**Fig. 6:**
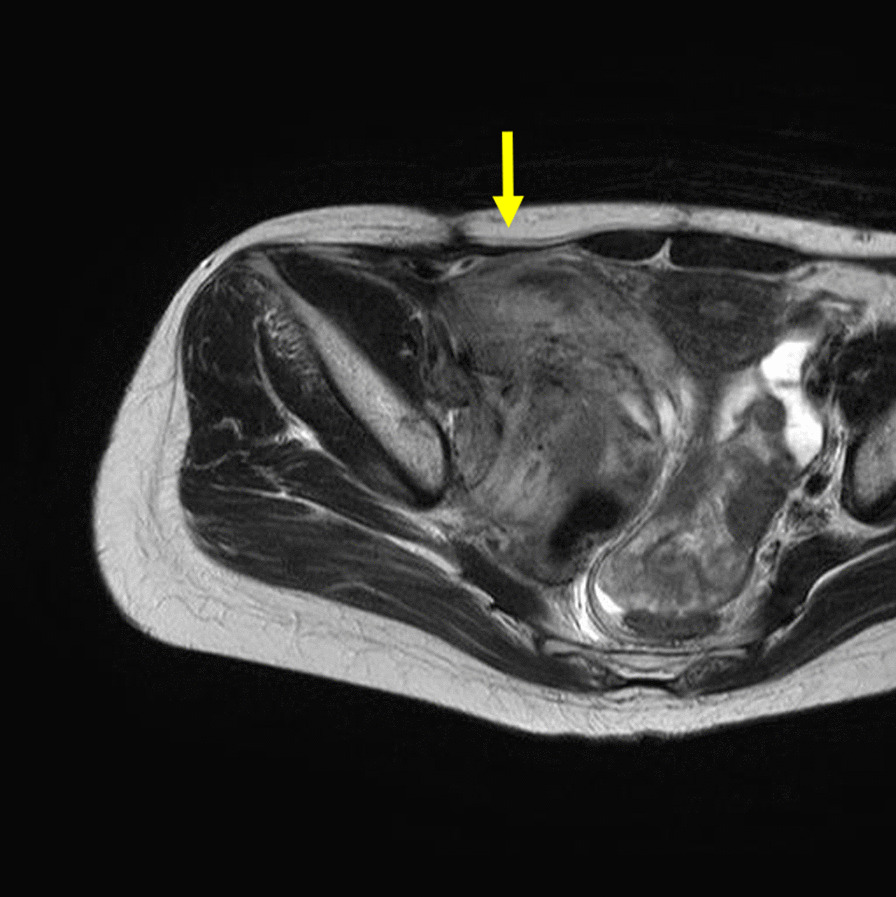
Axial T2-weighted image of the pelvis at 1 month after radiotherapy. Temporary tumor progression is seen (yellow arrow).

**Fig. 7:**
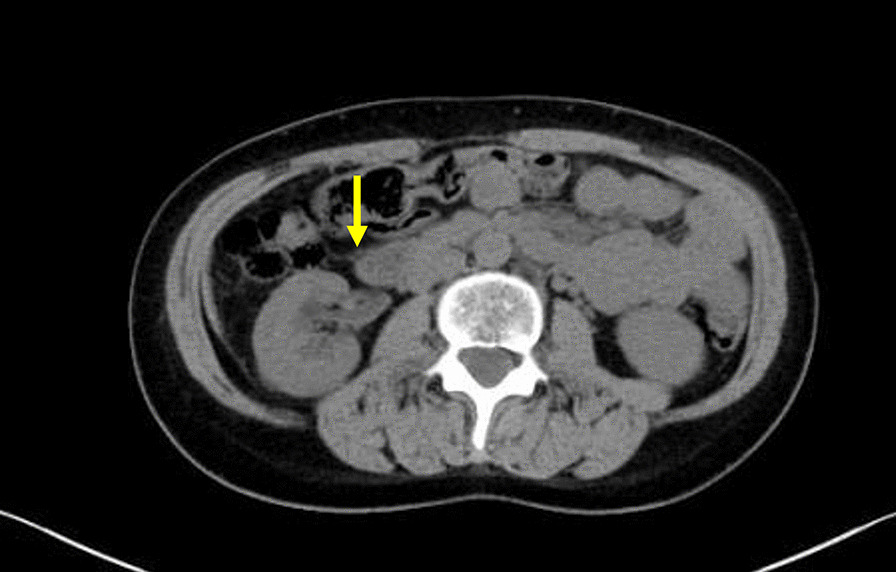
Axial computed tomography scan image of the right kidney 2 years after radiotherapy. Hydronephrosis improved (yellow arrow)

**Fig. 8:**
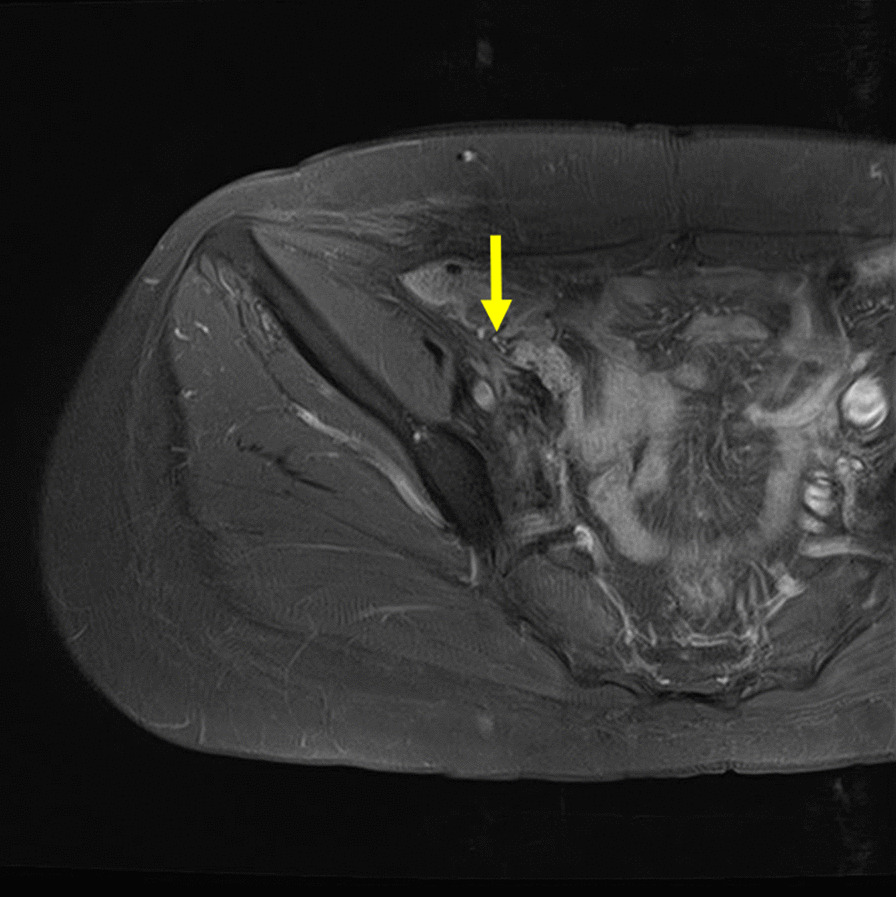
Post-gadolinium axial magnetic resonance imaging of pelvis. The lesion in the right pelvic wall shows no marked enhancement by intravenous administration of gadolinium (yellow arrow)

At 1 month after RT, there was temporary tumor progression and the tumor was 7.9 × 10.5 cm in size (Fig. 6). However, on subsequent follow-up visits, slow regression of the tumor was seen. The tumor had almost disappeared 2 years after RT. Hydronephrosis also improved (Fig. 7). At 5 years after RT, which was 10 years after surgery, there was no disease progression (Fig. 8). The timeline for intervention and clinical outcome is presented in Table 1.

**Table 1:** Timeline

The event	Timeline (months)
Initial symptom in right inguinal region in the 15th week of pregnancy	*T* = 0
Radiological investigations (MRI: suggestive of fibromatosis)	*T* = 1
Fine needle aspiration biopsy (diagnosis of DTF)	*T* = 2
Resection of the initial DTF in the 18th week of pregnancy	*T* = 3
Follow-up MRI (diagnosis of recurrence DTF)	*T* = 39
Cyclooxygenase-2 selective inhibitor treatment	*T* = 40
Follow-up CT (increase in the size of recurrence DTF and diagnosis of hydronephrosis)	*T* = 51
Radiation therapy for recurrence DTF	*T* = 53
No signs of recurrence or no adverse events	*T* = 103

*DTF* desmoid-type fibromatosis, *CT* computed tomography, *MRI* magnetic resonance imaging

## Discussion and conclusions

Abdominal wall DTF has been shown to have a relatively good outcome. Crago *et al*. reported that 5-year local recurrence-free survival rate after surgery for abdominal wall DTF was 91% [9]. However, abdominal wall DTF can result in hydronephrosis. Ureteral stenting and renal fistula creation are common treatments for hydronephrosis-associated malignant extrinsic ureteral obstruction (MUO), but their indications are controversial, especially for long survivors. Although stenting is a noninvasive treatment for hydronephrosis, it causes local discomfort related to urination. In addition, a renal fistula causes a decrease in quality of life (QOL) due to physical activity restriction by a drainage tube. Considering the patient’s QOL, it is often difficult to decide whether to perform ureteral stenting or renal fistula creation [10]. According to previous reports, hydronephrosis associated with DTF occurred in 28% of patients with intraabdominal DTF, and these cases required a ureteral stent or renal fistula [4]. Treatment of MUO should be judged from the primary tumor, renal function, patient and family wishes, symptoms associated with ureteral obstruction, presence or absence of chemotherapy, and prognosis [10]. In our case, the reason why stenting was not insisted upon was that renal function was relatively good. However, previous reports on the relationship between stent placement and renal function suggest that stent failure may occur at 4–5 mg of creatinine [10, 11]. The renal function in our case was not in a state of immediate stenting, and radiation therapy was not likely to reduce renal function. Horan *et al*. also reported that pelvic radiation therapy did not induce any deterioration in renal function or degree of hydronephrosis [12].

We were able to treat DTF with hydronephrosis by radiation therapy without surgical treatment or ureteral stent insertion, which the patient rejected. Our results indicated that radiation therapy without stenting for hydronephrosis in a patient with normal renal function is one of the options for treatment of DTF. A previous study showed that medication therapy alone for DTF with hydronephrosis was successful [13]. As far as we know, our case is the first reported case of long-term successful control of DTF with hydronephrosis by radiation therapy alone.

In our case of abdominal wall DTF, one of the causes of recurrence was considered to be that RT could not be performed after the initial surgical treatment despite the presence of a positive margin because the patient selected to continue her pregnancy. The clinical behavior of DTF in females who are pregnant is not clear. Marco Fiore reported that DTF progression during or after pregnancy can usually be safely managed by watchful waiting alone [14]. Another study showed that pregnancy does not increase the local recurrence rate after surgical resection of DTF [15]. Considering these reports, the postoperative course of DTF in our case is different.

Additionally, conservative management has recently been recommended. Retrospective studies have shown that patients can respond to drug therapy and that spontaneous regression can occur after recurrence [16, 17], but the size of the tumor increased despite drug therapy in our case. Another study showed that there was a tendency for a lower local control rate in patients with a larger number of operative procedures before RT and in patients who had been treated for recurrent aggressive fibromatosis [18]. Therefore, in pretreatment evaluation, it was considered that our case might be resistant to RT. Contrary to pretreatment predictions, the success of our treatment may have been due to the dose of irradiation. Regarding the dose of RT for DTF, there seems to be a general consensus for postoperative irradiation and irradiation for unresectable primary cases, but treatment for cases of recurrence in which postoperative irradiation could not be performed is unclear.

There have been few reports with detailed description of the method of RT for progressive recurrent DTF. Several studies have indicated that 50 Gy is sufficient for postoperative patients [19, 20].Therefore, the initial RT in our case was performed with 50.4 Gy for the right inguinal region. Retrospective studies have shown that irradiation at 36–75 Gy is a common treatment dose for DTF and that a dose above 56 Gy is a risk for complications in inoperable cases [6, 20, 21]. Considering these reports, it is notable that our unique point was to use a combination of wide-field RT and local RT to increase the dose. As a result, we could irradiate the DTF recurrence with a total dose of 59.4 Gy, and a good therapeutic effect was obtained with few complications. On the other hand, irradiation of a wide field can result in an increase in the risk of secondary cancer. When we treated our patient, intensity-modulated radiation therapy (IMRT) was not used for pelvic tumors in our institution. Although IMRT has excellent dose localization, it may also increase the risk of secondary cancer in long-term survivors [22].

We experienced successful treatment for a case of progressive recurrent DTF with unilateral hydronephrosis that could not be sufficiently treated initially because the patient was pregnant. Despite the difficulty of continuing conservative treatment after surgery due to tumor progression and onset of hydronephrosis, prevention of recurrence and successful local control without severe complications were achieved in our case for 5 years after RT alone with a moderate dose.

## Data Availability

The data include individual patient data, but the data are available from the corresponding authors upon reasonable request.

## Abbreviations

DTF: Desmoid-type fibromatosis
Gy: Gray
IMRT: Intensity-modulated radiation therapy
MRI: Magnetic resonance imaging
MUO: Malignant extrinsic ureteral obstruction
MV: Megavolt
QOL: Quality of life
RT: Radiation therapy
